# Electromyography Biomarkers for Quantifying the Intraoperative Efficacy of Deep Brain Stimulation in Parkinson's Patients With Resting Tremor

**DOI:** 10.3389/fneur.2020.00142

**Published:** 2020-02-26

**Authors:** Kai-Liang Wang, Mathew Burns, Dan Xu, Wei Hu, Shi-Ying Fan, Chun-Lei Han, Qiao Wang, Shimabukuro Michitomo, Xiao-Tong Xia, Jian-Guo Zhang, Feng Wang, Fan-Gang Meng

**Affiliations:** ^1^Department of Functional Neurosurgery, Beijing Neurosurgical Institute, Capital Medical University, Beijing, China; ^2^Department of Neurology, Fixel Center for Neurological Diseases, Program in Movement Disorders and Neurorestoration, University of Florida, Gainesville, FL, United States; ^3^Beijing Key Laboratory of Neurostimulation, Beijing, China; ^4^Department of Neurosurgery, Beijing Tiantan Hospital, Capital Medical University, Beijing, China; ^5^Department of Neurosurgery, General Hospital of Ningxia Medical University, Yinchuan, China

**Keywords:** Parkinson, DBS, efficacy quantifying, resting tremor, EMG

## Abstract

**Introduction:** Deep brain stimulation (DBS) is an effective therapy for resting tremor in Parkinson's disease (PD). However, quick and objective biomarkers for quantifying the efficacy of DBS intraoperatively are lacking. Therefore, we aimed to study how DBS modulates the intraoperative neuromuscular pattern of resting tremor in PD patients and to find predictive surface electromyography (sEMG) biomarkers for quantifying the intraoperative efficacy of DBS.

**Methods:** Intraoperative sEMG of 39 PD patients with resting tremor was measured with the DBS on and off, respectively, during the intraoperative DBS testing stage. Twelve signal features (time and frequency domains) were extracted from the intraoperative sEMG data. These sEMG features were associated with the clinical outcome to evaluate the efficacy of intraoperative DBS. Also, an sEMG-based prediction model was established to predict the clinical improvement rate (IR) of resting tremor with DBS therapy.

**Results:** A typical resting tremor with a peak frequency of 4.93 ± 0.98 Hz (mean ± SD) was measured. Compared to the baseline, DBS modulated significant neuromuscular pattern changes in most features except for the peak frequency, by decreasing the motor unit firing rate, amplitude, or power and by changing the regularity pattern. Three sEMG features were detected with significant associations with the clinical improvement rate (IR) of the tremor scale: peak frequency power (*R* = 0.37, *p* = 0.03), weighted root mean square (*R* = 0.42, *p* = 0.01), and modified mean amplitude power (*R* = 0.48, *p* = 0.003). These were adopted to train a Gaussian process regression model with a leave-one-out cross-validation procedure. The prediction values from the trained sEMG prediction model (1,000 permutations, *p* = 0.003) showed a good correlation (*r* = 0.47, *p* = 0.0043) with the true IR of the tremor scale.

**Conclusion:** DBS acutely modulated the intraoperative resting tremor, mainly by suppressing the amplitude and motor unit firing rate and by changing the regularity pattern, but not by modifying the frequency pattern. Three features showed strong robustness and could be used as quick intraoperative biomarkers to quantify and predict the efficacy of DBS in PD patients with resting tremor.

## Introduction

Parkinson's disease (PD) is a progressive neurodegenerative disorder characterized by certain typical motor symptoms: resting tremor, rigidity, and bradykinesia (1–3). In recent years, deep brain stimulation (DBS) has been established as an effective treatment for PD, especially for the motor symptoms. DBS modulates the basal ganglia circuits through high-frequency electrical stimulation (4). The most commonly used and approved targets for PD-DBS include the subthalamic nucleus (STN), the globus pallidus internus (GPi) and the ventral intermediate (VIM). Other DBS experimental targets include the pedunculopontine nucleus (PPN), nucleus of the thalamus, the caudal zona incerta (cZI), centromedian, and parafascicular nuclei (CMPf) (5, 6). Neurosurgeons and the neurologist together choose the optimal targets according to the symptoms that primarily affect the patient's life and how the patient responded to DBS therapy. To achieve optimal stimulation efficacy, the targets for some patients would be changed one, two, or even more times with rescue or replacement operations or with repeat multiple-pass mapping through the use of microelectrode recording during the DBS testing stage (7–9). Furthermore, the currently established evaluation methods for DBS efficacy mostly depend on the neurosurgeon's or neurologist's experience, intraoperative patients' self-response, and post-operative assessment of a clinical scale (UPDRS, Unified Parkinson's Disease Rating Scale) (10, 11). Two major weak points of this evaluation system are the subjectivity of symptom assessment and the time delay before the surgery for target replacement (7, 12). Therefore, an intraoperative and objective evaluation method is crucial for determining the efficacy of DBS in PD patients with resting tremor.

More and more studies have indicated that PD patients show aberrant kinetic functioning patterns of discharge in motor units (MUs), which can be measured by surface electromyography (sEMG) (13–17). sEMG signals are usually considered as an accumulation effect of activated MUs under the electrodes and are often used to evaluate the activity of the neuromuscular system. Therefore, sEMG enables the objective quantification of neuromuscular function and movement and could be adopted as a biomarker to assess the disrupted neuromuscular system in PD patients and even the treatment effect of DBS and drugs (14). As previous studies have reported, DBS might exert its therapeutic action through altering sEMG characteristics like amplitude, duration, domain tremor frequency of muscular burst, rhythmicity or regularity, and tremor-electromyogram coherence (18, 19). However, all of these quantitative metrics are measured a long time after DBS operation and not during the operation. Therefore, no intraoperative biomarker for quantifying and predicting the efficacy of DBS in PD patients with resting tremor is available.

The purpose of the current study is to investigate how and to what extent the neuromuscular pattern of resting tremor in PD patients could be modulated by intraoperative DBS through measuring sEMG characteristics. Furthermore, given the lack of quick biomarkers, we also aimed to explore robust sEMG biomarkers for quantifying and predicting intraoperative DBS efficacy.

## Subjects and Methods

### Study Subjects

As one of the three cardinal clinical symptoms in PD, resting tremor is the most common symptom and the easiest to observe. It is also the symptom that responds the most rapidly to DBS treatment and is easy to observe, usually within several seconds to a few minutes. In contrast, the effects of DBS treatment on rigidity and bradykinesia are more difficult to observe. Both should be evaluated over the long term with regular DBS programming and are not easy to assess quickly during the operation (17, 20). Therefore, resting tremor could be a suitable clinical symptom for measuring the acute effects of the intraoperative DBS treatment. Furthermore, good tremor control is also the minimum aim for DBS in PD patients.

In the current study, we included PD patients with visible resting tremor as study subjects. Thirty-nine patients (22 male and 17 female; aged 60.51 ± 8.96 years, mean ± SD) with visible resting tremor were enrolled in the study. The patients were evaluated by a multidisciplinary team at Beijing Tiantan Hospital, as shown in Table 1. The diagnosis of advanced PD was based on clinical criteria (21, 22). Informed written consent was obtained from all patients, and all procedures were approved by the ethics committee and the neuromodulation committee at Beijing Tiantan Hospital.

**Table 1:** Demographic and clinical information of patients.

**Variable**	**Value (Mean ± SD)**
Number of patients	39
Gender (male/female)	22/17
Course of disease (Years)	8.26 ± 6.17
Age at surgery (Years)	60.51 ± 8.96
Hoehn-Yahr stage (Number)	
2	2
2.5	14
3	19
4–5	4
DBS target (STN/GPi)	35/4

*GPi, globus pallidus interna; STN, subthalamic nucleus*.

The DBS surgical procedures were performed in three stages (23): the (i) electrode implantation stage, (ii) quick testing stage, and (iii) implantable pulse generator (IPG) implantation stage. For the first stage (i), the electrodes were implanted under local anesthesia using the Leksell Stereotactic System (Elekta Instrument AB, Sweden). Intraoperative single-unit recordings were used to localize the motor subregion of the chosen target. Permanent quadripolar electrodes (3,387 Medtronic, Minneapolis, MN, USA for the GPi and 3,389 Medtronic, Minneapolis, MN, USA for the STN) were positioned in the motor subregions of the respective stimulation targets. We then performed the intraoperative quick testing stage (ii) to examine the efficacy of DBS treatment. Usually, we adopted an initial DBS programming setting of voltage (0.5 V), frequency (130 Hz), and pulse width (PW 90 μs). The frequency and the pulse width were fixed, and the voltage was increased to 5 V with a step size of 0.1 V until the optimal balance between symptom control and side effects was achieved. Two DBS-experienced neurologists evaluated the quick intraoperative clinical efficacy of DBS stimulation. Once satisfactory efficacy was achieved, the third stage (iii) was started. Otherwise, the stimulation coordinates of the targets were modified (or other targets were tested). For the third stage, the electrodes were connected to an IPG implanted in the subclavicular area under general anesthesia. After that, post-operative CT was performed to exclude intracranial hemorrhage and to verify the exact location of the electrodes through the fusing of the CT images with the preoperative MR images. The IPG was turned on 1 month after the operation, and DBS programming was followed. All post-operative adjustments of DBS parameter settings were performed while subjects were in an off-medication state.

### sEMG and Clinical Outcome Measurements

During the second stage (ii) of intraoperative testing, the sEMG was recorded using a Nicolet multiparameter electrophysiological instrument (Nicolet Corporation, Madison, Wisconsin, USA) with a sampling rate of 512 Hz. Bipolar electrodes (one over the belly of the muscle and one for the tendon with an at least 3 cm inter-electrode spacing) were attached to the limbs with obvious tremor symptoms using disposable Ag/AgCl electrodes as described in previous studies (12, 17): the extensor digitorum and the flexor digitorum superficialis for the forearms and the tibialis anterior muscle and the gastrocnemius muscle for the lower legs.

During all phases of sEMG recording, the patients' extremities with tremor were kept in an absolutely relaxed and resting position, fully supported against gravity. Continuous sEMG data were measured at two time-points during the quick testing stage (ii). The first one was the baseline (DBS-off state, after DBS lead implantation) sEMG data and was recorded before the DBS was turned on, when the lead reached the pre-planning stimulation position. The second time point was the stimulation-on (DBS-on) sEMG data. The patient's symptoms changed when the stimulation voltage was increased. Once a satisfactory therapeutic effect was achieved and reported via the intraoperative patient's self-response and observation, rapid motion activity, or the neurologists' experience and when resting tremor could not be evoked by limb movements, the programming parameter setting was fixed. Then the patient's second sEMG was recorded. At least 60 s of stable sEMG was recorded for each trial.

The patients' clinical outcomes were assessed using the motor section (part III) of the Movement Disorder Society-sponsored revision of the Unified Parkinson's Disease Rating Scale (MDS-UPDRS) (10) and the tremor subscale (item 15–18) of the MDS-UPDRS 1 month after the DBS surgery when the stimulator was turned on. All the clinical outcomes were evaluated in the off-medication state.

### Pre-processing of Signals and Feature Analysis

**Figure 1:**
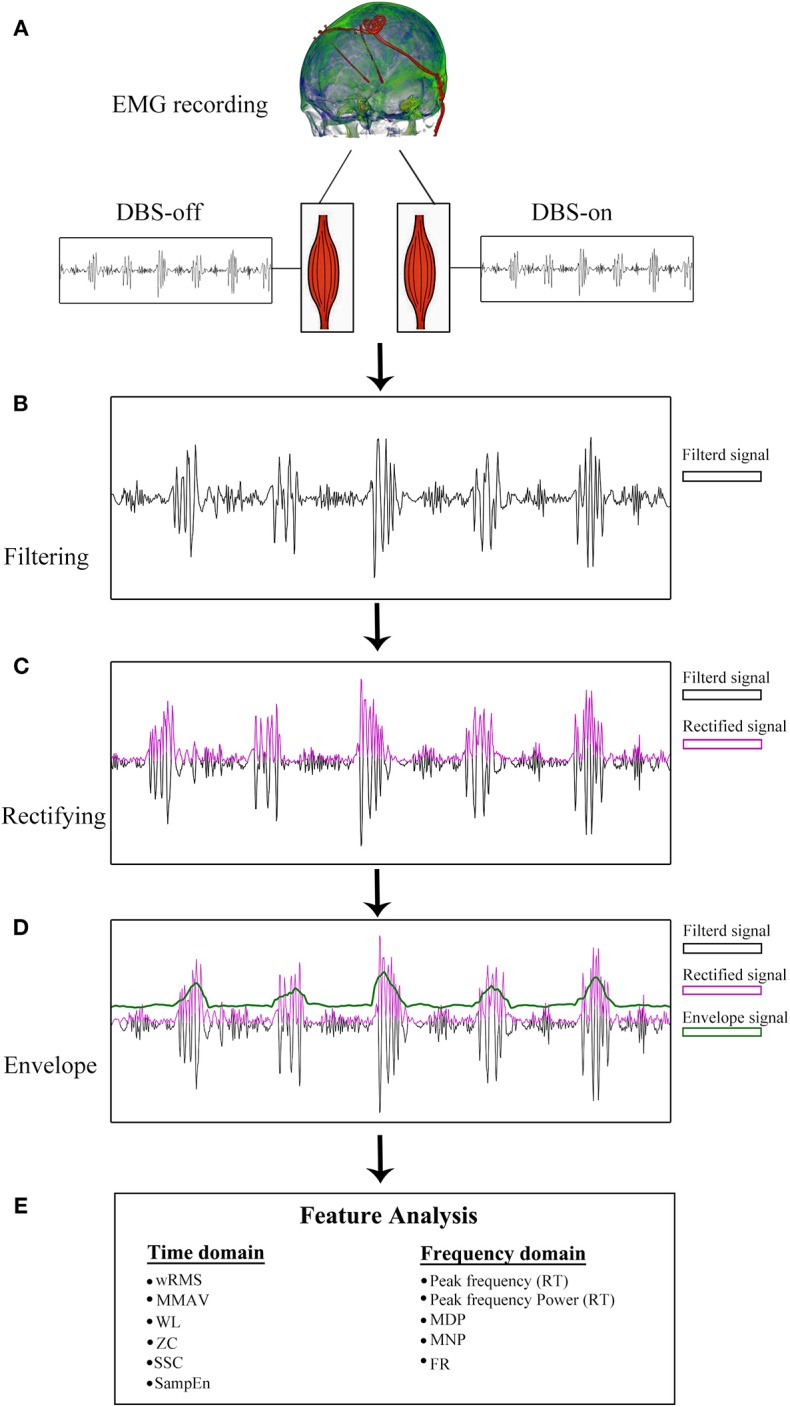
sEMG signal pre-processing diagram. **(A)** sEMG data recording with DBS-on and DBS-off. **(B)** Filtered signals between 20 and 200 Hz. With respect to the resting tremor (RT) burst detection, the filtered signals were full-wave rectified **(C)** and enveloped **(D)**. **(E)** Twelve features were analyzed based on pre-processed sEMG signals.

The sEMG data were pre-processed before feature computations. Briefly, all sEMG signals of PD patients were first visually inspected by two experienced sEMG experts to remove non-resting motor-related signals with high peaked artifacts. Second, the sEMG signal was band-pass filtered between 20 and 200 Hz with a self-adapting-order Butterworth filter. As recommended in previous studies (24), the sEMG signals were segmented into small 1 s segments and the features were averaged across all segments, as shown in Figure 1.

**Figure 2:**
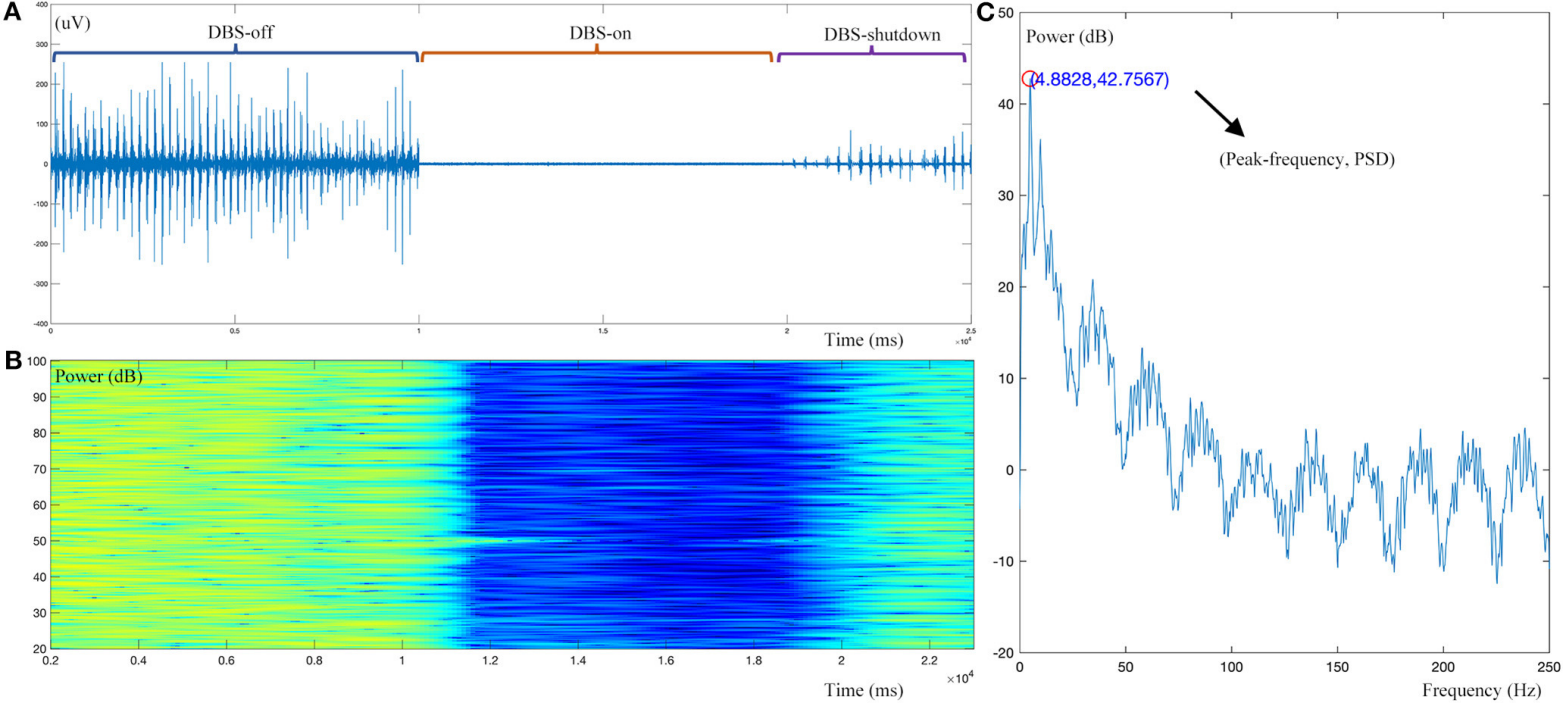
Example dynamic changes in the sEMG signal during DBS operation. **(A)** Raw sEMG data during the DBS-off, DBS-on, and DBS-shutdown phases. **(B)** Time-frequency analysis of the pre-processed sEMG signal. **(C)** Resting tremor (RT) burst detection, showing a peak frequency of 4.88 Hz and peak frequency power [Peak(f)PSD] of 32.76 dB. As shown, this case has a typical resting tremor frequency of 4.88 Hz. With intraoperative DBS on, time-frequency analysis demonstrated that the amplitude or power of resting tremor was obviously decreased.

For the tremor burst detection, the pre-processed sEMG signals were full-wave rectified to increase the signal-to-noise ratio (24). Since tremor was driven by rhythmic MU spike firing, the traditional Fourier-transform based methods were limited to extracting the burst signals directly (25, 26). Here, we adopted the Matlab built-in envelop function to detect the tremor burst, which searched for the root-mean-square envelopes of input signals, as shown in Figures 1, 2.

The following time-domain and frequency-domain features were measured from the pre-processed sEMG signals [the feature formulae have been described in detail in previous studies (27, 28)]. All signal analyses were performed using MATLAB (Math Works, Natick, MA, USA).

Time domain:

- Weighted Root Mean Square (wRMS): RMS value for each second (segment)
- Modified Mean Absolute Value (MMAV1 and MMAV2): estimated the mean absolute value of the sEMG
- Waveform Length (WL): measured the cumulative length of the waveform over the segment
- Zero Crossings (ZC): measured the times the waveform crosses zero, namely the number of times when the waveform changes its sign
- Slope Sign Changes (SSC): also measured the number of times the slope changed its sign
- Sample Entropy (SampEn) (14): measured the degree of rhythmicity or regularity of the sEMG signal, a measure of the time-dependent structure of the signal.

Frequency domain:

- Peak Frequency [Peak(f)]: measured the dominant peak frequency of the tremor burst based on P-welch estimation
- Peak Frequency Power [Peak(f)PSD]: measured the power spectral density of the Peak frequency based on P-welch estimation
- Median Amplitude Power (MDP): measured the median amplitude spectrum in each segment
- Mean Amplitude Power (MNP): measured the mean amplitude spectrum in each segment
- Frequency Ratio (FR): the ratio of the lowest frequency in a segment to the highest frequency in the segment.

The above-described 12 features (27) can be divided into three categories; those that measured the frequency [Peak(f), MU firing rate (FR, ZC, SSC)], the amplitude or power [wRMS, MMAV1, MMAV2, Peak(f)PSD, WL, MDP, MNP], or the regularity (SampEn) of the sEMG signal.

### Statistical Analysis

The basic characteristics and clinical outcome scores of PD patients were described as mean ± standard deviation (SD). The improvement rate (IR) was calculated between pre-operative scores and each post-operative follow-up as [100 [post-operative scores—pre-operative scores]/pre-operative scores]. Paired-*T*-tests were used to determine whether there was a significant difference between the clinical scale scores and sEMG features at baseline and stimulation-on. The robustness and inter-relationship between features were evaluated by co-correlation analysis based on Spearman correlation. Spearman correlation analysis was also employed to identify sEMG features associated with the tremor sub-scale of MDS-UPDRS.

The selected features that showed significant association with the tremor sub-scale of MDS-UPDRS were then used to create a machine-learning-based prediction model to predict the clinical tremor improvement with acute DBS stimulation. In general, datasets were divided into training sets and testing sets, which were optimized by a cross-validation algorithm using a leave-one-out cross-validation (LOOCV) iteration procedure to protect against model overfitting through partitioning of the data into folds (29–31). We then correlated the predicted values to the real IR of UPDRS-t and examined the correlation coefficient (*r*-value) and its significance. Furthermore, the significance of the prediction model established was tested through permutation testing (1,000 times). In brief, the PD patients' IR values of UPDRS-t were randomly permuted 1,000 times, and we compared the obtained *r*-value at each iteration with the true predictive *r*-value (true *r*-value). The number of permutations achieving a greater value than the true *r*-value was used to derive a *P*-value.

The statistical significance threshold was fixed at *p* < 0.05. Statistical analysis was performed with IBM SPSS (version 20.0; SPSS Inc, Chicago, IL, USA) and MATLAB (Math Works, Natick, MA, USA).

## Results

### Demographic Results

The demographic characteristics of the PD patients are described in Table 1. The course of the patients' disease was 8.26 ± 6.17 years, and most patients were in Hoehn-Yahr stage 2.5 (14/39) or 3 (19/39). At the time of the surgery, patients were 60.51 ± 8.96 years old. Thirty-five patients were stimulated at the STN and four patients at the GPi, as described in Table 1.

### Clinical Outcome

As measured by MDS-UPDRS (part III), the total motor score (off-medication UPDRS-III) was 63.42 ± 17.85 before the DBS stimulation, and the DBS-off sub-scale of the tremor part (off-medication UPDRS-t, items 15–18) was 15.60 ± 5.13 (mean ± SD). With stimulation on, the scores were significantly reduced to 31.19 ± 14.35 (UPDRS-III) and 3.71 ± 2.18 (UPDRS-t), respectively. For UPDRS-III, the improvement rate was 0.52 ± 0.15 (*p* < 0.0001), and for UPDRS-t, the improvement rate was 0.76 ± 0.15 (*p* < 0.0001), as described in detail in Table 2.

**Table 2:** Clinical scores and EMG features between intraoperative DBS-off and DBS-on in PD patients.

**Scores/features**	**DBS-off (Mean ± SD)**	**DBS-on**	**Improvement rate**
**Clinical scale**			
UPDRS-III	63.42 ± 17.85	31.19 ± 14.35	0.52 ± 0.15 (^*^)
UPDRS-t	15.60 ± 5.13	3.71 ± 2.18	0.76 ± 0.15 (^*^)
**EMG feature**			
**Frequency domain**			
Peak frequency	4.93 ± 0.98	4.77 ± 2.81	0.03 ± 0.56 (ns)
Peak frequency	44.57 ± 11.06	11.30 ± 14.54	0.79 ± 0.41 (^*^)
PSD			
MDP	204.07 ± 191.34	21.66 ± 42.72	0.87 ± 0.16 (^*^)
MNP	500.09 ± 4.19	476.04 ± 29.32	0.05 ± 0.06 (^*^)
FR	0.15 ± 0.06	0.12 ± 0.07	0.16 ± 0.46 (^*^)
**Time domain**			
wRMS	4.21 ± 3.98	0.46 ± 0.57	0.84 ± 0.17 (^*^)
MMAV1	32.73 ± 25.62	3.93 ± 5.59	0.85 ± 0.15 (^*^)
MMAV2	22.79 ± 18.30	2.83 ± 4.23	0.84 ± 0.84 (^*^)
WL	1565.10 ± 1869.91	125.81 ± 263.30	0.86 ± 0.30 (^*^)
ZC	9.69 ± 6.54	2.20 ± 3.45	0.71 ± 0.52 (^*^)
SSC	12.63 ± 8.71	3.31 ± 4.77	0.65 ± 0.60 (^*^)
SampEn	0.64 ± 0.30	1.12 ± 0.40	−1.31 ± 1.63 (^*^)

*The UPDRS-III and UPDRS-t were evaluated among 35 patients, and data for four patients were missing or not recorded*.

*GPi, globus pallidus interna; STN, subthalamic nucleus; ns, non-significant*,

* *Indicates p < 0.001*.

### sEMG Features

**Figure 3:**
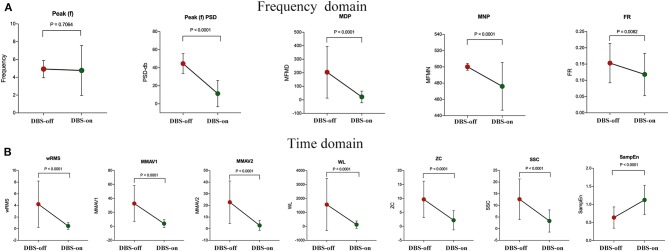
sEMG signal feature analysis between intraoperative DBS-off and DBS-on. **(A)** Features of the frequency domain and **(B)** features of the time domain. As shown, except for the feature of peak frequency (*p* = 0.7064), the features changed significantly with intraoperative DBS-on. Compared to the baseline (DBS-off), in the frequency domain, Peak(f)PSD, MDP, MNP, and FR all showed significantly decreased values after DBS stimulation. In the time domain, wRMS, MMAV1, MMAV2, WL, ZC, and SSC were significantly decreased as well. For the regularity analysis, the SampEn value was significantly increased with DBS-on.

Except for peak frequency (*p* = 0.7064), the intraoperative sEMG features were significantly changed during intraoperative DBS-on, as shown in Table 2 and Figure 3. Compared to the baseline, as to the amplitude or power-related features, wRMS, MMAV1, MMAV2, Peak(f)PSD, WL, MDP, and MNP all showed decreased values after DBS stimulation, indicating that DBS stimulation controlled the resting tremor through suppressing the aberrant tremor amplitude. For the regularity analysis, SampEn was significantly (*p* < 0.01) increased from 0.64 ± 0.30 (DBS-off) to 1.12 ± 0.40 (DBS-on), which demonstrated that the DBS-off regular (pathological) signals were restored to more random (normal) signals after intraoperative DBS stimulation.

For the features measuring the frequency characteristics (Peak(f), FR, ZC, and SSC), Peak(f) was used to assess the resting tremor frequency. The remaining features (FR, ZC, and SSC) were adopted to examine the related characteristics of the MU firing rate. Here, the detected resting tremor Peak(f) was 4.93 ± 0.98 Hz at the baseline (DBS-off) with a non-significant change with DBS stimulation (4.77 ± 2.81 Hz, *p* = 0.71). However, the features of FR, ZC, and SSC were significantly changed by DBS treatment. Tremor results from the accumulation effects of activated MUs. Therefore our results indicate that DBS only reduced the firing rate of the MUs but could not totally disrupt the pathological synchronization of the resting tremor in the MUs (i.e., DBS could not change the resting tremor frequency immediately or during the operation time without enough programming and stimulation time).

### Correlation Analysis of sEMG Features and Clinical Scales

**Figure 4:**
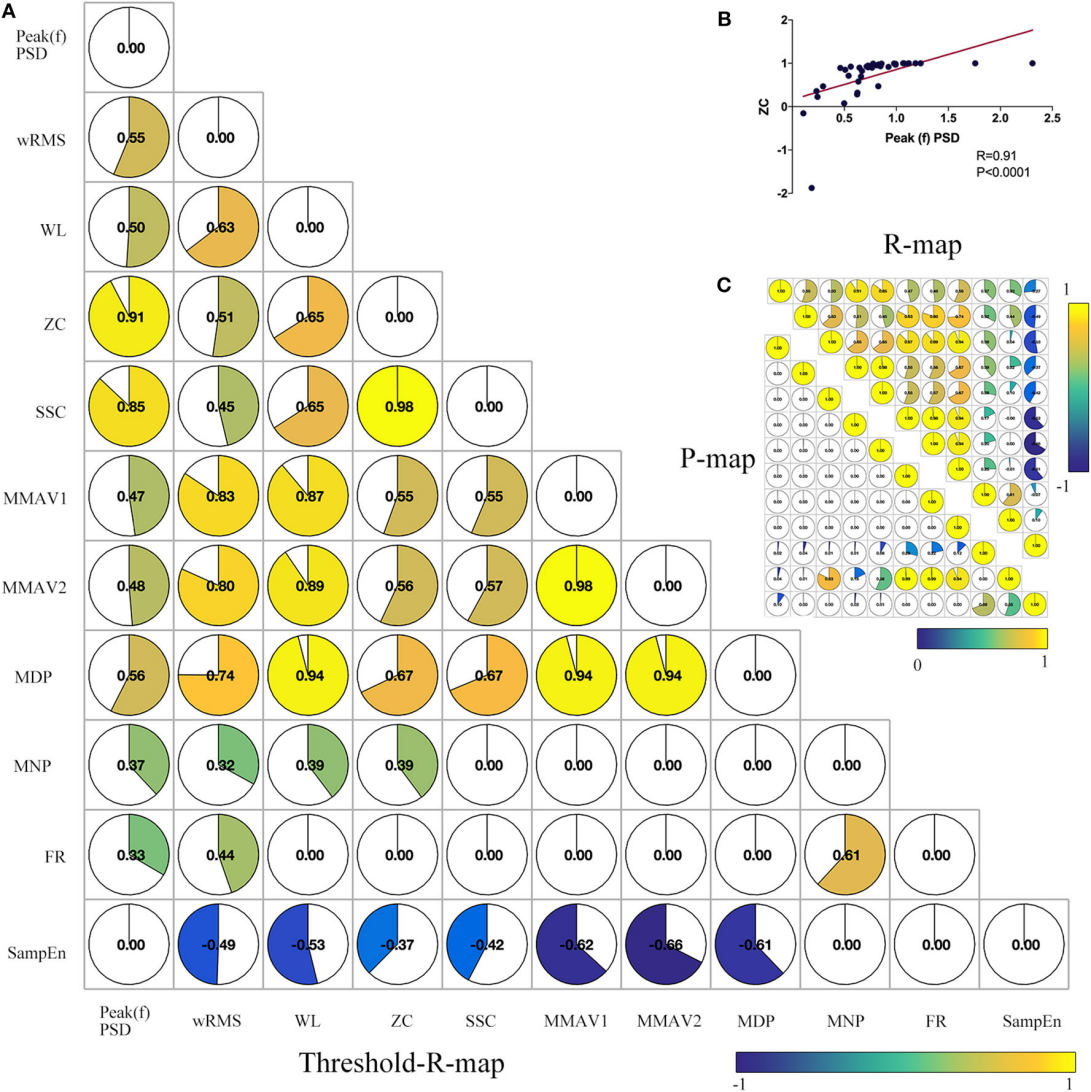
Co-correlation analysis between significant sEMG signal features. **(A)** Combined correlation R-map under the threshold of *p* < 0.05 (without multiple correction). The value in the matrix indicates the significant correlation *R*-value with inter-feature Spearman correlation. **(B)** Example co-correlation analysis between feature ZC and Peak(f)PSD. **(C)** The upper triangular matrix represents the raw correlation R map, and the lower triangular matrix demonstrates the raw correlation P map. As shown, wRMS has 10 co-correlated features. Peak(f)PSD, WL, and ZC have three co-correlated features. SSC, MMAV1, MMAV2, and MDP have eight co-correlated features. SampEn, MNP, and FR have seven, five, and three co-correlated features, respectively.

To test the robustness or reduce the redundancy or overlapping effects of sEMG features and find inter-relationships between features, we performed a co-correlation analysis between sEMG features. Briefly, 11 significant features were correlated “feature to feature” through Spearman correlation, as described in Figure 4. The feature of wRMS significantly correlated with all of the remaining 10 features. As described in Figure 4, Peak(f)PSD, WL, and ZC had nine correlated features. However, FR only significantly correlated with three features. The features wRMS, Peak(f)PSD, WL, and ZC might contain more components of other features and might contribute a higher accuracy as EMG biomarkers, as they could be interpreted as more comprehensive. In contrast, FR, MNP, and the other features correlated with fewer remaining features, which might reflect more “specific” roles in the sEMG characteristics, which cannot be explained and replaced by the “comprehensive” features.

**Figure 5:**
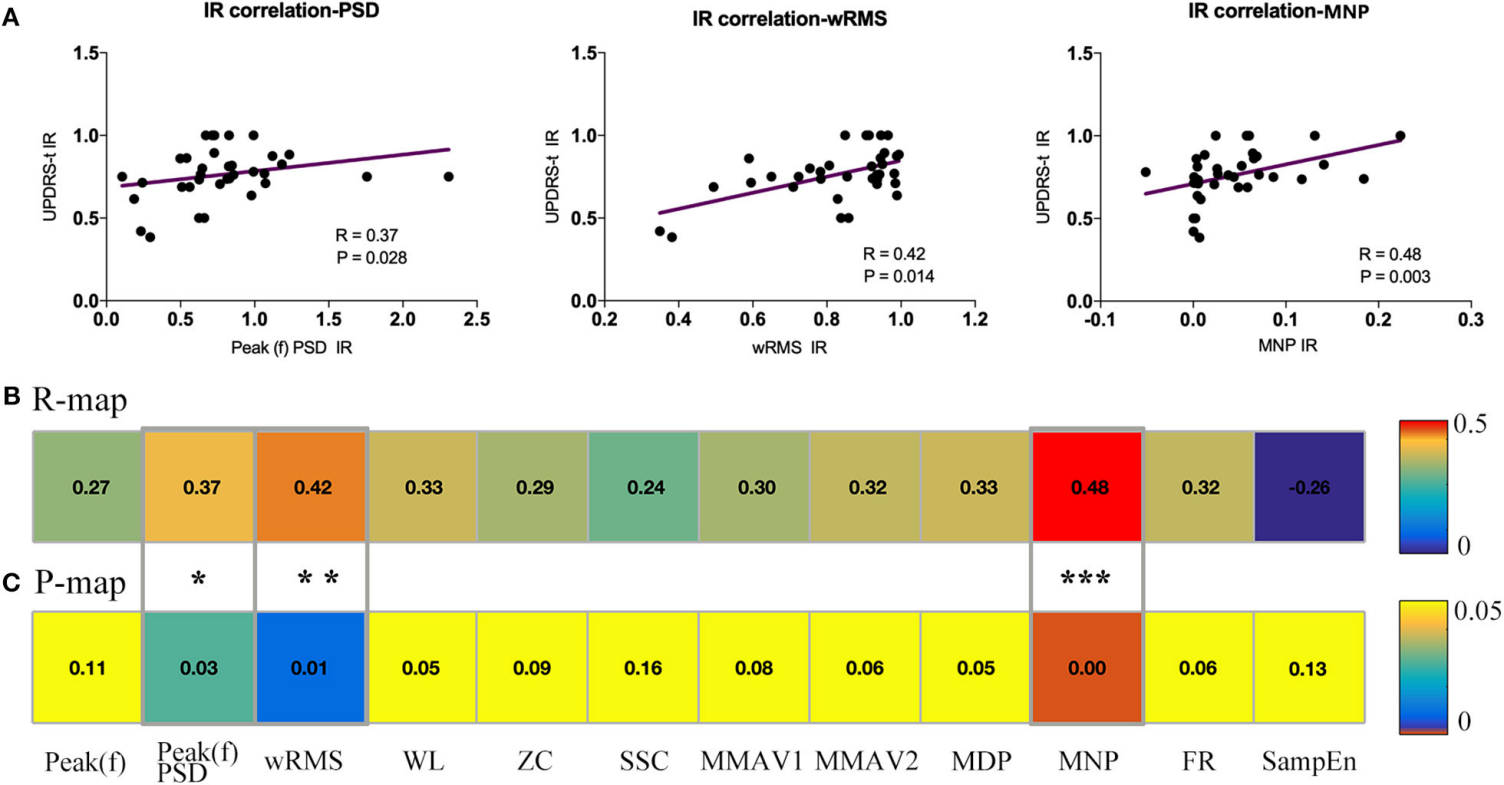
Association analysis between sEMG signal features and UPDRS-t. All changing 12 sEMG features were correlated with the clinical improvement rate (IR) of UPDRS-t. **(A)** Three important sEMG features were detected to have significant correlation with the IR of UPDRS-t, including Peak(f)PSD (*R* = 0.37, *p* = 0.03), wRMS (*R* = 0.42, *p* = 0.01), and MNP (*R* = 0.48, *p* = 0.003). All raw correlation analyses are shown in **(B)** R map and **(C)** P map. ^*^Indicates significant changes. ^**^, ^***^Indicates *p*-values.

It is well-established that feature reduction and selection is a very important procedure of model estimation for machine learning (32). To further explore the validity of all 12 sEMG features and find the most effective intraoperative biomarkers for quantifying the efficacy of DBS, we also performed correlation analysis between the improvement rate of the 12 sEMG features and the improvement rate of UPDRS-t. As described in Figure 5, Peak(f)PSD (*R* = 0.37, *p* = 0.03), wRMS (*R* = 0.42, *p* = 0.01), and MNP (*R* = 0.48, *p* = 0.003) showed a significant association with UPDRS-t.

Co-correlation analysis divided the 11 features into “specific” ones and “comprehensive” ones, and the association estimation between sEMG features and UPDRS-t detected three useful features among them for quantifying the intraoperative efficacy of DBS, namely Peak(f)PSD, wRMS, and MNP.

### sEMG Prediction Model and Prediction Results

**Figure 6:**
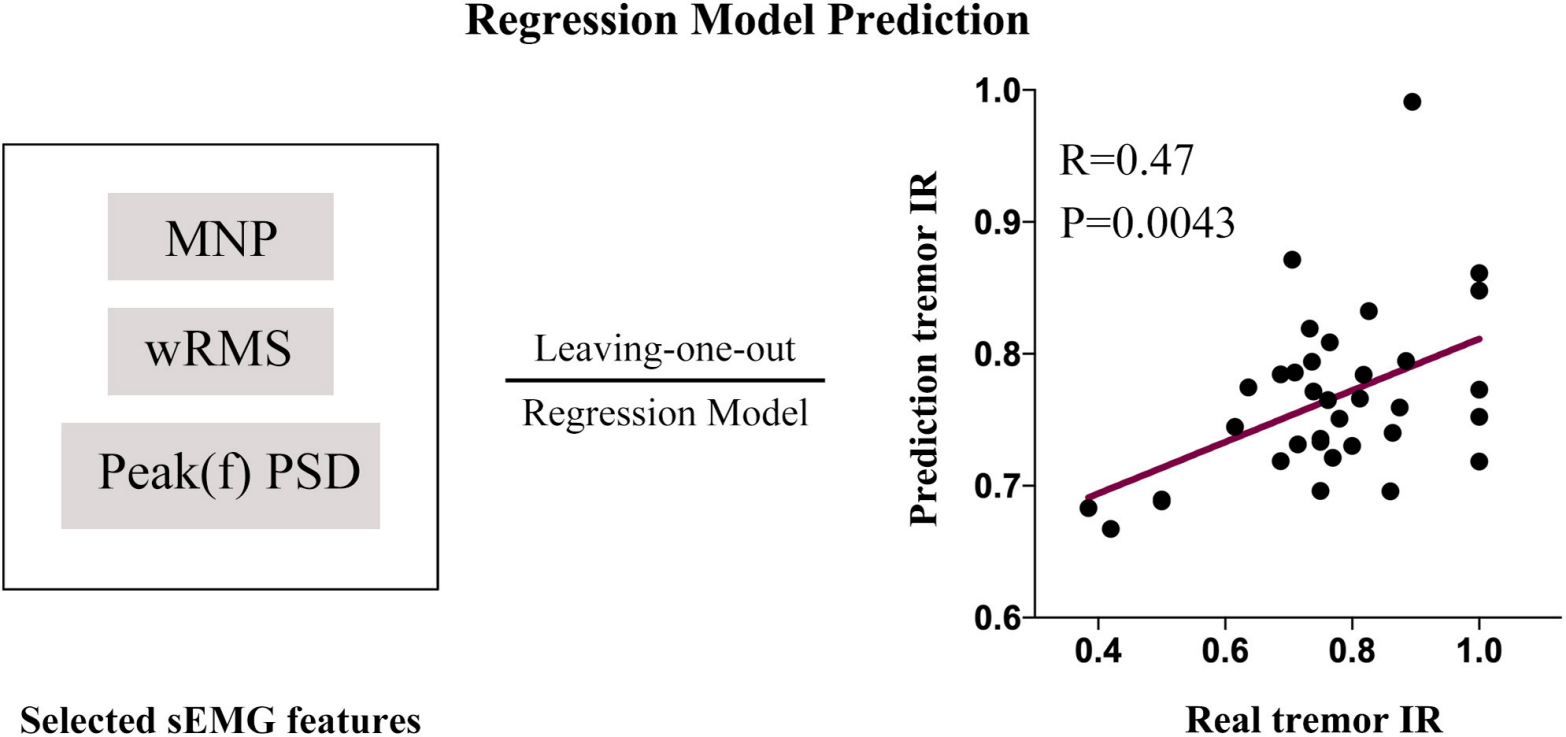
An sEMG feature (Peak(f)PSD, wRMS and MNP)-based prediction model was established with a leave-one-out cross-validation procedure. The model was trained with the Gaussian process regression (GPR) model (1,000 iterations of permutation testing, *p* = 0.003). The predictive IR value showed a significantly positive correlation with the real IR of UPDRS-t (*r* = 0.47, *p* = 0.0043).

The three selected sEMG features (Peak(f)PSD, wRMS, and MNP) were inputted into the Gaussian process regression (GPR) model (using the Regression Learner App built-in to Matlab) to train the sEMG prediction model with the cross-validation algorithm of LOOCV. The predictive IR value showed a significant positive correlation with the true IR of UPDRS-t (*r* = 0.47, *p* = 0.0043). With 1,000 iterations of permutation testing, the sEMG prediction model with three robust features achieved a significant *p*-value of 0.003. The sEMG prediction model and prediction results are described in Figure 6. The Matlab codes for the sEMG prediction model in the current study have been made publicly available: https://github.com/kailiang-wang/sEMG-model-for-DBS-PD.

## Discussion

The purpose of the current study was (i) to explore the underlying mechanism by which DBS modulates the intraoperative neuromuscular pattern of resting tremor in PD patients with resting tremor and (ii) to find effective sEMG biomarkers for quantifying and predicting the intraoperative efficacy of DBS. Using sEMG data of DBS-on and -off states from PD patients with resting tremor during the intraoperative testing stage, we analyzed 12 sEMG features measuring the frequency or MU firing rate (4/12), amplitude or power (7/12), and regularity (1/12) of the sEMG signal. We revealed three important findings regarding intraoperative DBS. First, DBS exerts its intraoperative therapeutic effect mainly by suppressing neuromuscular amplitude and regularity patterns but not frequency. Second, DBS did not change the resting tremor frequency immediately but reduced the MU firing rate. Third, three useful and quantitative sEMG biomarkers were detected; Peak(f)PSD, wRMS, and MNP showed a significant association with the clinical scale and could be considered as predictive biomarkers for detecting the intraoperative efficacy of DBS for resting tremor. Our results provide new evidence of quantifying and predicting the intraoperative efficacy of DBS by exploring sEMG biomarkers that could be used to aid clinical DBS treatment.

### sEMG Biomarkers for DBS

The assessment of the effectiveness of intraoperative DBS has always been challenging for DBS experts, although it is critical for surgical decision-making. Traditional subjective evaluation methods of repetitive passive movements or patient self-response are not sufficiently sensitive and precise to assess the real therapeutic effects in a variety of clinical settings (12). Therefore, biomarkers to represent and quantify the expected response need to be defined. In the past few years, sEMG has been proposed to discriminate patients from normal controls (14, 33). It was also used to distinguish between essential tremor and tremor of PD through spectrally based methods, such as amplitude analysis (14), wavelet-based approaches (34, 35), linear and non-linear parameters (33, 36), EMG-burst shape analysis (36, 37), and a principal component approach (12).

However, to our knowledge, only five studies have investigated sEMG biomarkers for quantifying the efficacy of DBS, as described in previous studies (12, 17, 36, 38). In line with previous studies, our results confirmed that sEMG features were sufficiently robust and sensitive to quantify the efficacy of DBS via three sEMG biomarkers: Peak(f)PSD, wRMS, and MNP. In addition, the present study includes the largest sample size for an sEMG study in DBS patients to date. On the other hand, the previous sEMG studies on DBS did not focus on the intraoperative assessment of the effects of DBS but tested the effects in post-operative patients, who might have been stimulated for a long time with proper programming and some of whom even showed a stable therapeutic effect. Our study, however, was designed to study DBS intraoperatively in a clinical setting. Compared to long-term DBS PD patients, the efficacy of DBS in intraoperative patients would be more challenging to evaluate because these patients were receiving DBS stimulation for the first time. The therapeutic effect of intraoperative DBS might not be stable, especially for rigidity, bradykinesia, gait problems, and non-motor symptoms, which depend on the long-term application of DBS, and even show a long time delay effect (4). The clinical manifestation of a rapid response that is easy to observe and addresses the main symptoms patients complain about is vital for capturing the immediate effectiveness of DBS treatment in the environment of the operating theater. The remaining symptoms can be treated gradually with the optimal and long-term DBS programming settings (20). For this reason, here, we chose PD patients with obvious resting tremor and used resting tremor as the main metric to measure the efficacy of intraoperative DBS. Our study showed that the sEMG features of resting tremor are the first preliminary biomarkers to evaluate and predict the efficacy of intraoperative DBS, including Peak(f)PSD, wRMS, and MNP, especially for the prediction effects of these sEMG biomarkers, which have been rarely reported in previous studies.

### Mechanism of Intraoperative DBS Treatment for Resting Tremor

In the current study, our patients achieved a clinical improvement of 0.52 ± 0.15 UPDRS-III when the stimulation was on for the first time after the operation, which could be used to mimic the intraoperative stimulation. Our result was in line with the report in Kleiner-Fisman et al. (39) that an improvement of 52% was achieved based on 37 cohort studies. Hence, the intraoperative effectiveness of DBS in our study is as effective as in other studies (33–35). We also used items 15–18 of UPDRS-III to define the sub-scale of tremor and measured a tremor improvement of 0.76 ± 0.15 (*p* < 0.001). Although we only recorded and assessed the sEMG data based on the resting tremor, the total motor and tremor symptoms were both controlled well in our patients. Thus, the benefit of using intraoperative sEMG biomarkers for quantifying the efficacy of DBS for resting tremor is also supported by the correlation analysis between sEMG features (Peak(f)PSD, wRMS, and MNP) and the tremor sub-scale of UPDRS-III (UPDRS-t).

The mechanisms responsible for the modulation of resting tremor by DBS remain unclear (2). Circuit models of the basal ganglia might provide a possible explanation (5). STN and GPi are both important nodes of this model, and stimulating both targets would change the neural activity, which is correlated with tremor, as suggested by animal models (40, 41) and functional connectome analysis (42–44). In the current study, three sEMG features were detected that were correlated to tremor modulation, namely Peak(f)PSD, wRMS, and MNP, which all measured the amplitude or power of tremor. The Peak(f)PSD measured the power of the typical resting tremor of 4.93 ± 0.98 Hz (45, 46), while wRMS and MNP evaluated the muscular power of the whole frequency band. Although the MU firing rate and regularity of resting tremor also changed, neither was found to be significantly associated with the UPDRS-t improvement rate. One reason for this could be that intraoperative DBS has an immediate effect but that long-term (chronic) stimulation would produce a positive and significant effect on muscular activity and resting tremor. Thus, our study showed that only the amplitude or power feature could be considered as sEMG biomarkers for quantifying the intraoperative efficacy of DBS in PD patients with resting tremor.

There are several limitations to the present study. First, we did not include normal healthy people (NHP) as a control group to explore differences between PD patients and NHP or DBS-on patients and NHP. Furthermore, only intraoperative PD patients were studied. However, this was related to the purpose of our research, which was aimed at finding individual intraoperative sEMG biomarkers for quantifying the intraoperative efficacy of DBS instead of identifying PD patients from the normal population (33). In the future, an NHP group could be enrolled to study whether DBS restored the pathologic PD neuromuscular state to a normal state. Second, we only focused on the symptom of resting tremor to simplify the study design and find quick sEMG biomarkers, as mentioned above. However, as the results of UPDRS-III show, other symptoms were also controlled well in the current study. Future work will aim to find more sEMG biomarkers for quantifying and predicting the symptoms of rigidity, bradykinesia, and even non-motor symptoms for DBS-PD patients with chronic stimulation. Thirdly, although we recorded the sEMG data after the microlesioning effect had occurred, the clinical improvement of sEMG features with DBS-on might still mix with the microlesioning effect (47). However, it is still valid because the microlesioning effect could be considered a “stimulation” effect of intraoperative DBS, which often showed a “stimulation” effect with transient microhemorrhage, edema, gliosis proliferation, and synaptic plasticity from the stimulation tissue, which has already been proved to help predict motor benefit from DBS (47, 48).

## Conclusion

In summary, for the first time, intraoperative sEMG biomarkers were studied for quantifying and predicting the intraoperative efficacy of DBS in PD patients with resting tremor. Three important sEMG biomarkers were reported: Peak(f)PSD, wRMS, and MNP. On the other hand, DBS played an acute role in modulating the intraoperative resting tremor, mainly through suppressing the amplitude, regularity, and MU firing rate pattern rather than the frequency pattern. The current study provides new evidence to elucidate a potential mechanism of DBS treatment for intraoperative PD and found three useful sEMG biomarkers for quantifying the clinical success of DBS.

## Data Availability Statement

The raw data supporting the conclusions of this article will be made available by the authors, without undue reservation, to any qualified researcher.

## Ethics Statement

The studies involving human participants were reviewed and approved by the medical ethics committee of IRB of Beijing Tiantan Hospital Affiliated to Capital Medical University. The patients/participants provided their written informed consent to participate in this study.

## Author Contributions

FW, K-LW, and F-GM: study concept and design. S-YF, QW, DX, C-LH, and SM: data collection. K-LW, MB, S-YF, WH, and X-TX: analysis and interpretation. K-LW: drafting of the manuscript. F-GM, MB, WH, and FW: critical revision of the manuscript. FW, F-GM, and J-GZ: study supervision.

### Conflict of Interest

The authors declare that the research was conducted in the absence of any commercial or financial relationships that could be construed as a potential conflict of interest.
